# Genetic Association of Drug Response to Erlotinib in Chinese Advanced Non-small Cell Lung Cancer Patients

**DOI:** 10.3389/fphar.2018.00360

**Published:** 2018-04-11

**Authors:** Cong Wang, Fang Chen, Yichen Liu, Qingqing Xu, Liang Guo, Xiaoqing Zhang, Yunfeng Ruan, Ye Shi, Lu Shen, Mo Li, Huihui Du, Xiaofang Sun, Jingsong Ma, Lin He, Shengying Qin

**Affiliations:** ^1^Life Science College, Anhui Medical University, Hefei, China; ^2^Bio-X Institutes, Key Laboratory for the Genetics of Developmental and Neuropsychiatric Disorders (Ministry of Education), Shanghai Jiao Tong University, Shanghai, China; ^3^The Fourth Hospital of Jinan City, Taishan Medical College, Jinan, China; ^4^Department of Pharmacy, Shanghai Pulmonary Hospital, Tongji University School of Medicine, Shanghai, China; ^5^The Third Affiliated Hospital, Guangzhou Medical University, Guangzhou, China; ^6^Shanghai Center for Women and Children's Health, Shanghai, China

**Keywords:** non-small cell lung cancer, erlotinib, single-nucleotide polymorphism (SNP), therapeutic responses, adverse drug responses

## Abstract

The efficacy of erlotinib treatment for advanced non-small cell lung cancer (NSCLC) is due to its action as an epidermal growth factor receptor tyrosine kinase inhibitor (EGFR-TKI). Patients treated with erlotinib experience different drug responses. The effect of germline mutations on therapeutic responses and adverse drug responses (ADRs) to erlotinib in Chinese patients requires elucidation. Sixty Han Chinese advanced non-small cell lung cancer patients received erlotinib monotherapy and, for each participant, 76 candidate genes (related to EGFR signaling, drug metabolism and drug transport pathways) were sequenced and analyzed. The single-nucleotide polymorphisms (SNPs) rs1042640 in *UGT1A10*, rs1060463, and rs1064796 in *CYP4F11*, and rs2074900 in *CYP4F2* were significantly associated with therapeutic responses to erlotinib. Rs1064796 in *CYP4F11* and rs10045685 in *UGT3A1* were significantly associated with adverse drug reaction. Moreover, analysis of a validation cohort confirmed the significant association between rs10045685 in *UGT3A1* and erlotinib adverse drug response(unadjusted *p* = 0.015). This study provides comprehensive, systematic analyses of genetic variants associated with responses to erlotinib in Chinese advanced non-small cell lung cancer patients. Newly-identified SNPs may serve as promising markers to predict responses and safety in erlotinib-treated advanced non-small cell lung cancer patients after chemotherapy doublet.

## Introduction

Lung cancer is the leading cause of cancer-associated death worldwide (Jemal et al., 2011), of which approximately 85% is classified as non-small cell lung cancer (NSCLC). In China, more than 500 thousand deaths each year are attributed to NSCLC, with incidence rising at a rate of 26.9% (Ferlay et al., 2015; Torre et al., 2015). A high proportion of patients are diagnosed with locally advanced disease or distant metastases, making them ineligible for surgery. In these patients, prognosis for radiotherapy or chemotherapy is unsatisfactory with unfavorable side effects, resulting in a 5-year survival rate of less than 17% (Siegel et al., 2013; Canada, 2015). Thus, emergence of effective strategies for the treatment of NSCLC is urgently needed.

The last decade has witnessed significant progress in terms of understanding the biology and genomics of lung cancer. In recent years, novel therapeutic strategies developed in NSCLC that target specific genomic mutations have been firmly established and show promising efficacy. A large body of literature has demonstrated that aberrant epidermal growth factor receptor (EGFR) pathway activity is a backbone of NSCLC cancerogenesis, playing a considerable role in processes decreased in malignancies, such as cell proliferation, differentiation and migration (Bardelli et al., 2003; Sawyers, 2003; Ciardiello and Tortora, 2008). Epidermal growth factor receptor-tyrosine kinase inhibitors (EGFR-TKIs) were developed to treat patients with *EGFR* somatic activating mutations; they were first discovered to underpin NSCLC responsiveness to gefitinib in 2004 (Lynch et al., 2004; Mayo et al., 2012).

Erlotinib, a first generation EGFR-TKI, is economical and efficacious for advanced NSCLC patients. Pivotal trials of erlotinib demonstrated meaningful improvements in both progression-free survival (PFS) and overall response rate (ORR) compared with the standard platinum-doublet chemotherapy in *EGFR* mutant NSCLC patients (Hirsch et al., 2011; La Salvia et al., 2017; Yoshida et al., 2017). The BR21 study confirmed that erlotinib increased overall survival (OS) in patients who were unsuitable for other treatments, with strong efficacies in East Asians, non-smokers and patients with lung adenocarcinomas (Florescu et al., 2008). While EGFR-TKI significantly prolong OS in advanced NSCLC patients, real-world clinical evidence demonstrated considerable individual variability in both therapeutic efficacy and safety signals (Riely et al., 2009; Gainor and Shaw, 2013). Previous studies suggested that patients with deletions in *EGFR* exon 19 or point mutations in the L858R residue in exon 21 responded well to TKI therapy, whereas patients with the secondary T790M mutation were resistant to first-generation agents (Han et al., 2011). A wealth of additional studies published worldwide, have indicated that mutations in *KRAS, BRAF, ALK, RET, ROS1*, and *PIK3CA* influence EGFR-TKI sensitivities in lung adenocarcinomas (Takeuchi et al., 2012; Gainor and Shaw, 2013). Additionally, *KRAS* mutations may associate with EGFR-TKI resistance in NSCLC patients (Pao et al., 2005). *EGFR* and *KRAS* mutations showed mutual gene exclusion in a series of NSCLC patients, which agreed with these previous findings (Shigematsu et al., 2005).

In addition to *EGFR* gene mutations, expression levels of genes involved in enzymatic drug metabolism, drug transport, and intracellular signaling have correlated with erlotinib efficacy and ADR. Both erlotinib and gefitinib are transported by the ATP-binding cassette family proteins, ABCB1, and ABCG2, and then metabolized in the liver by cytochrome P450 (CYP450) and the UDP glucuronosyltransferase (UGT) family (Han et al., 2007; Kim et al., 2010). Activities of these gene products determine pharmacokinetics of the agents and therefore influence ADR and therapeutic response rates. Harmsen et al. reported that gefitinib- and erlotinib-induced ABCB1 increase activated accumulations of ABCB1 substrates *in vitro*, suggesting that ABCB1 influenced drug resistance in these models (Harmsen et al., 2013). Another study showed that reduced *CYP2D6* activity associated with high frequencies of skin rash in patients undergoing gefitinib therapy (Suzumura et al., 2012). *UGT* is one of vital roles in constitutive cellular metabolic pathways. Inter-individual variability in UGT pathway activities have been associated with drug metabolism and diseases (Suzuki and Sugiyama, 2002; Na et al., 2017).

Previous studies have focused on analyzing associations between candidate genes and drug responses in NSCLC; most studies emphasized somatic variants and failed to systematically and comprehensively identify and validate germline candidate biomarkers (Schmid et al., 2016). There is a pressing need to carry out non-biased studies identifying genetic variants in NSCLC that associate with responses to erlotinib and changes in safety signals. These data would guide clinical decision making when implementing personalized medicine. Here, single-nucleotide polymorphisms (SNP) in the EGFR signal pathway, as well as the drug metabolism and transport pathways, were investigated with next-generation sequencing (NGS) technologies to identify SNPs associated with drug responses to erlotinib in Chinese advanced NSCLC patients. Application of multiple genomic alteration screens will allow for improved treatment planning. Novel molecular biomarkers identified here may be useful for future research of therapeutic strategies in NSCLC patients.

## Materials and methods

### Study design

The objective of this study was to discover the genetic variants associated with drug reactions of erlotinib of lung cancer patients in Chinese population. The whole study was designed to be conducted in two steps. In the first step, using NGS method, we systematically investigated the germ line genetic variants of 76 genes contributions to EGFR signaling, EGFR antagonist drug metabolism, or transport in discovery cohort (60 samples). In the second step, the candidate genetic variant identified in the first step were verified invalidation cohort (134 samples).

### Patient recruitment

Blood samples were collected from 60 Chinese Han patients who underwent erlotinib monotherapy after prior standard chemotherapy for advanced NSCLC at the Shanghai Pulmonary Hospital. The patients with complete clinical information, accurate pathological diagnosis and classification criteria and without other diseases were recruited. Patient clinical records were obtained, including: gender, age at presentation, smoking status, cancer diagnosis, pathologic stage, PFS, and ADR (e.g., skin rash and/or digestive tract injury). The EGFR of each patients have been sequenced by NGS and validated by SNapShot. All cases in study carry EGFR mutation and none of the genetic variants has been reported associated with drug response of advanced NSCLC, so the patients recruited in our study received erlotinib as second-line treatment. In the validation study, 134 Chinese erlotinib monotherapy-treated patients with advanced NSCLC following prior chemotherapy were enrolled. Clinical demographics are given in **Table 5**; allelic type and frequency are given in Tables 1, 2.

**Table 1:** Clinical characteristics of erlotinib-treated advanced NSCLC cohort.

**Characteristics**		**All patients (*N* = 60)**	**Therapeutic response**		**Adverse drug response**	
			**Responder (*N* = 15)**	**Non-responder (*N* = 45)**	**ADR (*N* = 13)**	**Non-ADR (*N* = 47)**
Age, years	Median (range)	59.5 (43–83)	61 (44–80)	59 (43–83)	59 (44–80)	60 (43–83)
Sex	Female	17 (28.3%)	7 (46.7%)	10 (22.2%)	7 (53.8%)	10 (21.3%)
	Male	43 (71.7%)	8 (53.3%)	35 (77.8%)	6 (46.2%)	37 (78.7%)
Tumor stage	Stage IIIa	13 (21.7%)	4 (26.7%)	9 (20.0%)	5 (38.5%)	8 (17.0%)
	Stage IIIb	6 (10.0%)	3 (20.0%)	3 (6.7%)	2 (15.4%)	4 (8.5%)
	Stage IV	41 (68.3%)	8 (53.3%)	33 (73.3%)	6 (46.1%)	35 (74.5%)
Smoking status	Never	32 (53.3%)	7 (46.7%)	25 (55.6%)	8 (61.5%)	24 (51.1%)
	Ever	7 (11.7%)	3 (20.0%)	4 (8.9%)	3 (23.1%)	4 (8.5%)
	Current	21 (35.0%)	5 (33.3%)	16 (35.5%)	2 (15.4%)	19 (40.4%)
PFS, months	Median (range)	3.55 (0.3–29)	10 (7–9)	2 (0.3–6.2)	8.5 (0.5–29)	2 (0.3–18)

**Table 2:** Patient demographics in erlotinib-treated NSCLC validation cohort.

**Characteristics**		**All patients (*N* = 134)**	**Therapeutic response**		**Adverse drug response**	
			**Responder (*N* = 32)**	**Non-responder (*N* = 102)**	**ADR (*N* = 28)**	**Non-ADR (*N* = 106)**
Age, years	Median (range)	60 (39–83)	59.5 (44–80)	60 (39–83)	59 (45–80)	60 (39–83)
Sex (%)	Female	41 (30.6)	14 (43.8)	27 (26.5)	15 (53.6)	26 (24.5)
	Male	93 (69.4)	18 (56.2)	75 (73.5)	13 (46.4)	80 (75.5)
Tumor stage (%)	Stage III a	21 (15.7)	6 (18.8)	15 (14.7)	5 (17.9)	16 (15.1)
	Stage III b	15 (11.2)	5 (15.6)	10 (9.8)	2 (7.1)	13 (12.3)
	Stage IV	98 (73.1)	21 (65.6)	77 (75.5)	21 (75.0)	77 (72.6)
Smoking status (%)	Never	78 (58.2)	18 (56.3)	60 (58.8)	19 (67.9)	59 (55.7)
	Ever	16 (11.9)	5 (15.6)	11 (10.8)	1 (3.6)	14 (13.2)
	Current	40 (29.9)	9 (28.1)	31 (30.4)	8 (28.5)	32 (30.1)
PFS, months	Median (range)	4 (0.3–29)	10 (1–29)	2 (0.3–6.8)	4.6 (1–29)	4 (0.3–21)

Written informed consent was obtained from the participants in our study prior to enrollment. The Ethics Committee of Shanghai and the Ethical Committee of Human Genetic Resources approved the protocol used in this study. Patient recruiting, blood sample collection, and clinical data collection and usage were all performed according to the guidelines and regulations of the committees.

### Next generation sequencing

Seventy-six candidate genes were assessed. These genes were chosen due to their known contributions to *EGFR* signaling, *EGFR* antagonist drug metabolism, or transport in NSCLC. Candidate genes were submitted for HaloPlex probe design (Agilent Technologies, Santa Clara, CA, USA) using the Sure-Design service (Table 3) (https://earray.chem.agilent.com/suredesign/index.htm). Genomic DNA specimens were extracted from whole peripheral blood using AxyPrep Blood Genomic DNA Miniprep Kits (AxyGen, Shanghai, China) in strict accordance with standard protocols. The qualities and quantities of resultant DNA specimens obtained were measured with a NanoDrop 2000 spectrophotometer (Thermo Scientific, Commonwealth of Massachusetts, USA). DNA concentrations were adjusted to 20 ng/μl and stored at −20°C until use. Target regions were captured and enriched using HaloPlex Target Enrichment Kits (Agilent Technologies, Santa Clara, USA) according to the manufacturer's instructions. Libraries were pooled in equimolar aliquots. Amplicon sequencing reactions were performed at a 3 nM concentration per sample to counterbalance differences in sample quality. Sequencing was performed on an Illumina MiSeq benchtop sequencer (Illumina, San Diego, CA, USA). The variant call cutoff was 5%, and results were only interpreted if the coverage was >40x.

**Table 3:** Seventy-six candidate genes assessed by NGS.

**Candidate genes**										
**Drug transport**	***ABCB1***	***ABCG2***								
Drug metabolism	*CYP1A1*	*CYP1A2*	*CYP1B1*	*CYP2A13*	*CYP2A6*	*CYP2A7*	*CYP2B6*	*CYP2C8*	*CYP2C9*	*CYP2C18*
	*CYP2C19*	*CYP2D6*	*CYP2E1*	*CYP2F1*	*CYP2J2*	*CYP2R1*	*CYP2S1*	*CYP2W1*	*CYP3A4*	*CYP3A5*
	*CYP3A7*	*CYP39A1*	*CYP46A1*	*CYP4A11*	*CYP4A22*	*CYP4B1*	*CYP4F2*	*CYP4F3*	*CYP4F8*	*CYP4F11*
	*CYP4F12*	*CYP4F22*	*CYP4V2*	*CYP4X1*	*CYP4Z1*	*CYP7A1*	*CYP7B1*	*CYP8B1*	*CYP11B1*	*CYP11B2*
	*CYP17A1*	*CYP19A1*	*CYP20A1*	*CYP24A1*	*CYP26A1*	*CYP26B1*	*CYP26C1*	*CYP27A1*	*CYP27C1*	
	*UGT1A6*	*UGT1A8*	*UGT1A10*	*UGT2A1*	*UGT2A3*	*UGT2B4*	*UGT2B7*	*UGT2B10*	*UGT2B11*	*UGT2B15*
	*UGT2B17*	*UGT2B28*	*UGT3A1*	*UGT3A2*						
Tumor target	*EGFR*	*ALK*	*MET*	*TGF*	*TP53*	*HIF1A*	*APC*	*KRAS*	*BRAF*	*RET*
	*PIL3CA*	

### Statistical analyses

Raw data from successful sequencing runs were analyzed using Sure-Call software (version 2.0). SNP sites with success rates <90%, Minor allele frequency (MAF) <1%, or those that were homogeneous across all samples were excluded. The Response Evaluation Criteria in Solid Tumors system (RECIST guidelines version 1.1) was used to classify responses as CR, PR, SD, or PD. Associations were identified between patient genotypes and objective responses to erlotinib within the first month of medication. To analyze ADR, patients were subcategorized according to clinical incidence of treatment-related adverse events.

Hardy-Weinberg genetic equilibriums, allelic and genotypic frequencies, odds ratios (ORs) and 95% confidence intervals (CIs), SNP case-control association analyses and linkage disequilibrium analyses were performed using SHEsis software (http://analysis.bio-x.cn/myAnalysis.php.). *P*-values < 0.05 were considered statistically significant. Correlations between objective responses to EGFR-TKI and ADR, as well as multiple logistic regression analyses were calculated using SPSS software (version 18.0) (http://www-01.ibm.com/software/analytics/spss/). In validation trials, genotypic data were analyzed using SHEsis software and statistical analyses were performed using SPSS. *P*-values of two-tailed tests < 0.05 were considered statistically significant.

## Results

### Clinical characteristics

The clinical characteristics of the first discovery cohort are described in Table 1. NSCLC patients who showed complete response or partial response (PR) were considered erlotinib sensitive-responders and patients who showed progressive disease (PD) or stable disease (SD) were considered erlotinib-resistant non-responders (RECIST guidelines version 1.1).

The cohort consisted of 43 males and 17 females, 32 never-smokers, seven ever-smokers and 21 currently-smoking patients. Among the 60 patient-cohort, the median PFS was 3.55 months, ranging from 0.3 to 29 months. Fifteen patients were responders with a median PFS of 12.57 months. Forty-five patients were non-responders, with a median PFS of 2.7 months. Thirteen patients presented with adverse drug reactions. The most common adverse event was skin rash, which correlated with therapeutic response (Table 1). No significant differences were observed between responders and non-responders in characteristics including: sex, age and smoking status; indicating that the populations were adequately matched.

### SNP associated with objective responses to erlotinib

Four SNP sites, all from drug metabolism genes, associated with objective therapeutic responses to erlotinib (Table 4). Namely, rs1042640 (located in *UGT1A10*; *p* = 0.009), rs1060463 and rs1064796 (located in *CYP4F11*; *p* = 0.001 and 0.013, respectively), and rs2074900 (located in *CYP4F2*; *p* = 0.001) associated with therapeutic responses to erlotinib. Interestingly, the rs1060463 SNP in *CYP4F11* (a C>T point mutation) had the weakest association with objective therapeutic response. However, none of the associations between these SNP and response rates remain statistically meaningful after multiple-testing corrections.

**Table 4:** SNP sites and genotypes associated with drug responses.

**Classification**	**Allele number (frequency)**		***P-*value**	**Odds ratio [95%CI]**	**Genotype number (frequency)**			***P-*value**
*UGT1A10* rs1042640	C	G			C/C	C/G	G/G	
response	14 (0.500)	14 (0.500)	0.220915	1.709677 [0.721-4.053]	5 (0.357)	4 (0.286)	5 (0.357)	0.009243
non-response	31 (0.369)	53 (0.631)			15 (0.357)	1 (0.024)	26 (0.619)	
*CYF4F11* rs1060463	C	T			C/C	C/T	T/T	
response	13 (0.542)	11 (0.458)	0.001049	0.190616 [0.067-0.542]	5 (0.417)	3 (0.250)	4 (0.333)	0.001277
non-response	62 (0.861)	10 (0.139)			26 (0.722)	10 (0.278)	0 (0.000)	
*CYP4F11* rs1064796	C	G			C/C	C/G	G/G	
response	12 (0.462)	14 (0.538)	0.013415	3.112782 [1.236-7.836]	4 (0.308)	4 (0.308)	5 (0.385)	0.005965
non-response	19 (0.216)	69 (0.784)			1 (0.023)	17 (0.386)	26 (0.591)	
*CYP4F2* rs2074900	A	G			A/A	A/G	G/G	
response	9 (0.409)	13 (0.591)	0.001422	5.835165 [1.837-18.539]	3 (0.273)	3 (0.273)	5 (0.455)	0.005422
non-response	7 (0.106)	59 (0.894)			0 (0.000)	7 (0.212)	26 (0.788)	

### SNP associated with erlotinib-induced adverse events

Two SNP associated with adverse drug reactions (Table 5). Specifically, rs1064796 in *CYP4F11* (a G>C point mutation; *p* = 0.003) and rs10045685 in *UGT3A1* (an A>G point mutation; *p* = 0.017) associated with decreased risk of ADR. Interestingly, one SNP was associated with both response rates to erlotinib and improved safety signals. The rs10045685 mutation in the *UGT3A1* 3′untranslated region (UTR) significantly associated with both therapeutic responses and reduced ADR after erlotinib treatment. However, the associations between the A>G mutant allele and lowered risk of ADR were not found after either Bonferroni or false discovery rate (FDR) corrections.

**Table 5:** SNP sites and genotypes associated with drug adverse responses.

**Classification**	**Allele number (frequency)**		***P*-value**	**Odds ratio [95%CI]**	**Genotype number (frequency)**			***P*-value**
*UGT3A1* rs10045685	A	G			A/A	A/G	G/G	
ADR	9 (0.409)	13 (0.591)	0.016794	0.314685 [0.119-0.832]	4 (0.364)	1 (0.091)	6 (0.545)	0.071726
non-ADR	55 (0.688)	25 (0.312)			23 (0.575)	9 (0.225)	8 (0.200)	
*CYP4F11* rs1064796	G	C			C/C	C/G	G/G	
ADR	10 (0.455)	12 (0.545)	0.003496	4.133333 [1.536-11.121]	5 (0.455)	2 (0.182)	4 (0.364)	0.002551
non-ADR	62 (0.775)	18 (0.225)			2 (0.050)	14 (0.350)	24 (0.600)	

### Validation of associations between SNP and erlotinib responses

Validation experiments were conducted in 134 NSCLC patients who received erlotinib monotherapy; clinical information of the cohort is given in Table 2; allelic type and frequency are given in Table 6. Multiplex SNapShot assays were performed to confirm whether the five above-identified SNP significantly associated with erlotinib responses in this second, larger independent population. Results showed that rs10045685 (an A>G point mutation in the *UGT3A1* 3′UTR) associated with erlotinib-induced adverse responses (unadjusted *p* = 0.015; Table 7).

**Table 6:** SNP sites and genotypes associated with drug responses in NSCLC verification cohort.

**Classification**	**Allele number (frequency)**		***P*-value**	**Odds ratio [95%CI]**	**Genotype number (frequency)**			***P*-value**
*UGT1A10* rs1042640	C	G			C/C	C/G	G/G	
response	59 (0.922)	5 (0.078)	0.243088	1.80000 [0.663-4.887]	27 (0.844)	5 (0.156)	0 (0.000)	0.488198
non-response	177 (0.868)	27 (0.132)			77 (0.755)	23 (0.225)	2 (0.020)	
*CYP4F11* rs1060463	C	T			C/C	C/T	T/T	
response	37 (0.578)	27 (0.422)	0.306209	1.343761 [0.762-2.369]	9 (0.281)	19 (0.594)	4 (0.125)	0.230687
non-response	103 (0.505)	101 (0.495)			28 (0.275)	47 (0.461)	27 (0.265)	
*CYP4F11* rs1064796	C	G			C/C	C/G	G/G	
response	32 (0.500)	32 (0.500)	0.126967	0.645161 [0.367-1.135]	6 (0.188)	20 (0.625)	6 (0.188)	0.115721
non-response	124 (0.608)	80 (0.392)			39 (0.382)	46 (0.451)	17 (0.167)	
*CYP4F2* rs2074900	A	G			A/A	A/G	G/G	
response	15 (0.234)	49 (0.766)	0.433666	0.770584 [0.401-1.481]	1 (0.03)	13 (0.406)	18 (0.526)	0.324425
non-response	58 (0.284)	146 (0.716)			12 (0.118)	34 (0.333)	56 (0.549)	
*UGT3A1* rs10045685	A	G			A/A	A/G	G/G	
ADR	7 (0.125)	49 (0.875)	0.015167	0.361905 [0.155-0.844]	0 (0.000)	7 (0.250)	21 (0.750)	0.062029
non-ADR	60 (0.283)	152 (0.717)			10 (0.094)	40 (0.377)	56 (0.528)	
*CYP4F11* rs1064796	G	C			C/C	C/G	G/G	
ADR	22 (0.393)	34 (0.607)	0.537794	1.207792 [0.662-2.203]	10 (0.357)	14 (0.500)	4 (0.143)	0.791341
non-ADR	93 (0.493)	119 (0.561)			34 (0.321)	51 (0.481)	21 (0.198)	

**Table 7:** Validation of SNP associated with drug responses.

	**Gene**	**ID**	**Chromosome**	***P-*value**	**Effect**
Therapeutic response	*UGT1A10*	rs1042640	2	0.243088	UTR_3_PRIME
	*CYP4F11*	rs1060463	19	0.306209	NON_SYNONYMOUS
	*CYP4F11*	rs1064796	19	0.126967	SYNONYMOUS
	*CYP4F2*	rs2074900	19	0.433666	SYNONYMOUS
ADR	UGT3A1^*^	rs10045685	5	0.015167	UTR_3_PRIME
	*CYP4F11*	rs1064796	19	0.537794	SYNONYMOUS

* *significantly associated with drug response*.

### UGT3A1-miRNA functional predictions

To predict the function of *UGT3A1* in drug metabolism, an additional analysis was performed using the miRTarBase(http://mirtarbase.mbc.nctu.edu.tw/). *UGT3A1* is a target gene of the microRNA hsa-miR-181a-5p. This suggested that activity of this microRNA may related to responses to erlotinib treatment in *EGFR*-activated NSCLC. Moreover from The Cancer Genome Atlas (TCGA) database, hsa-miR-181a-5p was identified as an oncogene that targeted *UGT3A1* in lung adenocarcinomas.

## Discussion

EGFR-TKI treatment for NSCLC have an impressive profile of therapeutic responses and ADR compared with standard chemotherapies. Previous studies showed relationships between both somatic activating mutations in *EGFR* coding sequences and SNP of drug metabolism genes with therapeutic responses to EGFR-TKI (Ganzinelli et al., 2015; Lópezayllón et al., 2015; Ruan et al., 2016). For example, rs884225 in *EGFR* is associated with erlotinib-induced ADR. Given that drug responses have highly correlated with clinical outcomes, a systematic multi-gene approach was used to investigate genetic variations in therapeutic responses and ADR in erlotinib-treated advanced NSCLC Chinese Han patients. A significant association was identified between the SNP rs10045685 A>G in the 3′UTR of *UGT3A1* and reduced risk of erlotinib-induced ADR. Validation studies confirmed that rs10045685 associated with erlotinib-induced ADR, which might making this mutation a promising biomarker for responses to erlotinib in NSCLC patients.

Few previous studies have investigated *UGT3A1* variants in NSCLC. However, a recent study found that the *UGT3A* gene family catalyze biotransformation of endogenous and environmental carcinogens including drugs (Lu et al., 2015). Additionally, *UGT3A1* differentiated primate species by participation in sugar conjugation during metabolism and elimination of lipophilic metabolites and exogenous chemicals (Meech and Mackenzie, 2010). Also, increased presence of a *UGT3A1* inactivating mutations may have significant therapeutic and/or toxicological implications (Mackenzie et al., 2008). Therefore, the *UGT3A1* SNP rs10045685 may be a predictive molecular marker of improved erlotinib-induced ADR in NSCLC patients.

Additionally, the current study identified four SNP associated with objective responses to erlotinib: rs1042640 in *UGT1A10*, rs1060463, and rs1064796 in *CYP4F11* and rs2074900 in *CYP4F2*. Previous studies have shown that CYP450 family members function in cancer drug metabolism, including in NSCLC. Among CYPs, *CYP4F* subfamily is thought to be primarily involved in the metabolism of fatty acids and arachidonic acid metabolites (Dhar et al., 2008). To these authors' knowledge, associations between *CYP4F* expression and cancer have not been reported in any tumor type. Previous studies showed that increase of TNF-α-induced *CYP4F11* was in response to jun N-terminal protein kinase (JNK) pathway activity (Sechler et al., 2013). Another finding suggested that the CYP ω-hydroxylase, consisting of CYP4A and CYP4F molecules, promoted angiogenesis and metastatic potential in human NSCLC cells through up-regulation of Vascular endothelial growth factor (VEGF) and Matrix metallopeptidase 9 (MMP-9) (Wang et al., 2010; Bell and Strobel, 2012). Previously, *CYP2D6* was found to be another important CYP450 enzyme involved in drug responses, accounting for 30% of metabolism of all medications (Gurley et al., 2008). However, no significant associations were found, and the exact roles of *CYP2D6* in clinical settings were unclear (Guillemette et al., 2000; Mckillop et al., 2006). Additionally, *UGT1A7* contributed to detoxification by catalyzing the binding and subsequent deactivation of glucuronic acid with tobacco carcinogens, including: nitrosamines, benzopyrenes (Xiao et al., 2015; Rajappa et al., 2017). In contrast, mutations in *UGT1A10* associated with therapeutic responses to erlotinib in this study.

There were limitations to the current study. First, the blood samples tested were collected from a small cohort of patients that received erlotinib as second- or third-line therapy following chemotherapy (Rosell et al., 2012; Greenhalgh et al., 2015). First-line chemotherapy in *EGFR*-mutant NSCLC may decrease numbers of cancer cells, and diminish the overall clinical benefit of subsequent EGFR-TKI therapies. Second, most of the SNP associations identified in this study were not strong enough to persist after Bonferroni corrections. Given the strictness of multiple-testing corrections, type II error may have occurred when multiple sites were tested in these relatively small samples. Third, subcategorizing the patients into responder/non-responder, ADR/non-ADR is complicated clinically, which might have an effect on discovering genetic variants associated with TKI response. Also, false positive results could occur in small-samples studies, and further validation studies in larger samples should be conducted to verify whether erlotinib-induced ADR and therapeutic response had specific sets of predictive biomarkers and share common biomarkers.

In conclusion, in the present study we conducted a pilot study for high-throughput germline genetic variant analysis of drug response of NSCLC using second generation sequencing, which might provide potential candidate bio-targets for personalized medicine of erlotinib in clinic.

## Author contributions

Conception and design: SQ; Sample process: XZ, YL, FC, HD, and YS; Analysis: FC, YL, CW, QX, ML, LH, and YR; Interpretation of data: CW, FC, LG, LS, XS, HD, and YL; Drafting the article: CW, FC, YL, JM, and SQ. All authors contributed critical revision and final approval.

### Conflict of interest statement

The authors declare that the research was conducted in the absence of any commercial or financial relationships that could be construed as a potential conflict of interest.
